# Surgical Explantation of Transcatheter Aortic Valve Requires Patient-Tailored Management, but Challenges Remain

**DOI:** 10.3390/jcdd12100407

**Published:** 2025-10-15

**Authors:** Vasiliki Androutsopoulou, Andrew Xanthopoulos, Kalliopi Keramida, Arian Arjomandi Rad, Georgia Nazou, Prokopis-Andreas Zotos, John Skoularigis, Thanos Athanasiou, Dimitrios C. Iliopoulos

**Affiliations:** 1 Department of Cardiothoracic Surgery, University Hospital of Larissa, 41110 Larissa, Greece; zotospro@hotmail.com (P.-A.Z.); t.athanasiou@imperial.ac.uk (T.A.); 2 Department of Cardiology, University Hospital of Larissa, 41110 Larissa, Greece; andrewvxanth@gmail.com (A.X.); iskoular@med.uth.gr (J.S.); 3 Cardiology Department, General Anti-Cancer Oncological Hospital, Agios Savvas Athens, 11522 Athina, Greece; keramidakalliopi@hotmail.com; 4 Department of Cardiothoracic Surgery, Oxford Heart Centre, Oxford University NHS Foundation Trust, Oxford OX3 9DU, UK; arian.arjomandirad@medics.academy; 5 Department of Anesthesiology, Evangelismos General Hospital, 10676 Athens, Greece; georgianazou@gmail.com; 6 4th Cardiac Surgery Department, Hygeia Hospital, 15123 Athens, Greece; dciliop@yahoo.gr

**Keywords:** transcatheter aortic valve explantation, transcatheter aortic valve failure, surgical aortic valve replacement, transcatheter aortic valve replacement

## Abstract

Transcatheter aortic valve replacement has become a widely accepted alternative to surgical aortic valve replacement even in younger, lower-risk patients with longer life expectancy. The increasing use of transcatheter aortic valve replacement is leading to a rise in the need for surgical explantation of failing transcatheter valves, a complex procedure associated with increased periprocedural risks and technical challenges. The absence of established guidelines for the treatment of this life-threatening condition highlights an important clinical challenge. Published experience with surgical explantation of transcatheter aortic valve remains limited. Standardized explanation protocols, patient-tailored management, careful patient selection, detailed preoperative imaging, and early surgical referral are essential for improving safety and efficacy. Optimal outcomes require collaboration among cardiac surgeons, cardiologists, infectious disease specialists, and radiologists.

## 1. Introduction

Over the past twenty years, transcatheter aortic valve replacement (TAVR) technology has been undergoing development. TAVR is being increasingly considered as an alternative to surgical aortic valve replacement also in younger patients, most recently including selected individuals over 65 years of age, based on long-term data from the Partner 3 and Evolut Low Risk Trials [1,2].

As TAVR procedures are increasingly performed in younger and lower-risk patients, the number of surgical transcatheter aortic valve explants is expected to rise since there are several clinical scenarios in which surgical removal of a previously implanted transcatheter valve becomes necessary. Currently, the surgical replacement of a failing transcatheter aortic valve is one of the fastest-growing cardiac surgery procedures in the United States [3].

The annual incidence of TAVR explants is currently estimated at approximately 0.5% to 2%; however, this rate is anticipated to increase substantially in the future [3].

The surgical replacement of a failing transcatheter aortic valve is associated with increased periprocedural risk, reported median 30-day mortality between 15% and 20%, and requires specific technical skills and surgical experience. TAVR-explant procedures consistently demonstrated an observed-to-expected mortality ratio ranging from 1.5 to 3.5. The potential underestimation of risk scores may be attributed to factors such as a higher-risk patient population, increased clinical acuity, and procedural complexity. These considerations should be thoroughly evaluated when assessing candidates for TAVR explants.

**Figure 1:**
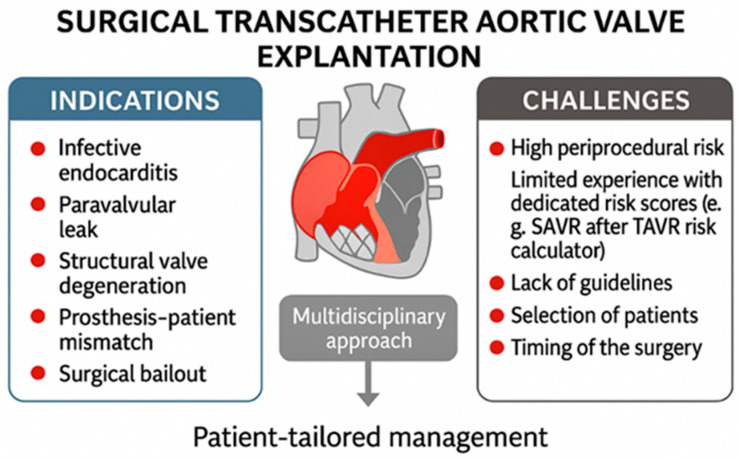
Challenges and indications of surgical transcatheter aortic valve explantation.

Currently, there are no established guidelines for the surgical management of patients undergoing open surgery in prior TAVR or standardized explanation protocols [3] [Figure 1].

## 2. Incidence and Indications for TAVR-Explant Surgery

According to the FRANCE 2 and FRANCE TAVI registries, which included 72,850 patients over an eight-year period, the cumulative incidence of overall reintervention in transcatheter aortic valve was 1.7%. Importantly, 62.1% of these reinterventions occurred early, within the first year following the initial procedure [4].

In the EXPLANT-TAVR international registry, which included 391 patients, the median time from TAVR to explantation was 13.3 months [5]. According to this registry, primary reasons for surgical removal are transcatheter valve endocarditis (43.1%), severe paravalvular leak (18.2%), structural transcatheter valve deterioration (20.1%), and severe prosthesis–patient mismatch (10.8%) [5,6] [Figure 1].

Surgical bailout is another indication for transcatheter valve explantation [Figure 1]. Specifically, urgent conversion from TAVR to open-heart surgery, referred to as surgical bailout, may be necessary in cases of complications such as annular rupture, coronary obstruction, ventricular perforation, subvalvular mitral valve apparatus impingement with the stiff guidewire, or type A aortic dissection. An analysis of the STS and ACC Transcatheter Valve Therapy registry found that, among 47,546 TAVR patients, 1.17% required surgical bailout, with an in-hospital mortality rate of 49.6% [7].

The cumulative incidence of transcatheter aortic valve endocarditis approaches 5% within the initial five years following the procedure. Reported annual incidence rates range from 0.5% to 1.6% among populations monitored for up to five years [8].

Recent analysis from the PARTNER-1 and PARTNER-2 trials, which included 8530 patients, revealed that the incidence of prosthetic valve endocarditis did not differ notably between patients who underwent TAVR and those who received surgical aortic valve replacement [9]. However, additional studies have highlighted the substantial morbidity and mortality associated with TAVR-related endocarditis. Notably, in-hospital mortality rates for TAVR-endocarditis were nearly twice as high as those observed in surgical prosthetic valve endocarditis cases [9].

Surgical explantation of infected TAVR prostheses is relatively rare, being performed in only 2% to 14% of cases despite clear indications for surgery in more than 80% of affected patients [6,8]. The primary reasons for not proceeding with surgery include high clinical or surgical risk, the need for additional procedures, advanced patient age, limited life expectancy, multiorgan failure, septicemia, and overall poor long-term prognosis. Unfortunately, this approach results in high in-hospital mortality rates and poor short-term survival. Among patients who undergo surgical explantation, postoperative in-hospital mortality has been reported to range from 28% to 47% [8,10].

## 3. EXPLANT-TAVR Registry: Comparison of Self-Expanding and Balloon-Expandable Valves

The EXPLANT-TAVR international multicenter registry includes patients who underwent surgical removal of a transcatheter aortic valve during a subsequent hospital admission. Between November 2009 and February 2022, a total of 391 patients underwent transcatheter aortic valve explantation across 42 centers [5]. This study systematically evaluates the outcomes and distinct clinical profiles of patients undergoing transcatheter aortic valve explantation due to failure of balloon-expandable valves (BEVs) compared to those with failed self-expanding valves (SEVs).

The study population had a mean age of 73.0 ± 9.8 years, with women comprising 33.8% of the cohort, independent of valve type. The median Society of Thoracic Surgeons (STS) risk score was 5.6%. Across all valve types, the median interval between implantation and explantation of the transcatheter aortic valve was 13.3 months (interquartile range: 5.1–34.8 months). The outcomes of 202 BEV procedures, representing 51.7% of the cohort, were compared with those of 189 SEVs, accounting for 48.3% [5].

This registry distinguishes between BEVs and SEVs, highlighting notable differences in the indications for explantation between the two device types. Indications for TAVR explantation included endocarditis, paravalvular leak, structural valve deterioration, and prosthesis–patient mismatch. The following table presents the distribution of common indications, along with their statistical significance as indicated by *p*-values [5] [Table 1].

Findings from the EXPLANT-TAVR registry show that, among patients with failed SEVs, there was a lower rate of urgent or emergency surgeries and a higher frequency of root replacement procedures compared to those with BEVs [5] [Table 2].

Concomitant cardiac procedures were conducted in 57.8% of patients, comprising coronary artery bypass grafting (24.8%), mitral valve surgery (38.9%), and tricuspid valve surgery (14.6%), without any significant differences observed between BEVs and SEVs [5]. Mitral surgery was the most frequently performed combined procedure and was identified as an independent predictor of mortality in both groups. Except for a higher rate of tricuspid valve surgery during SEV explantation (20.2% vs. 9.4%; *p* = 0.024), concomitant non-aortic procedures were similar between groups [5].

Patients with significant multivalvular disease needing mitral and/or tricuspid procedures had higher mortality at 30 days (23.5% vs. 9.4%, *p* = 0.006) and one year (46.0% vs. 22.1%, *p* = 0.002). They also faced longer bypass and ventilation times, more frequent pulmonary hypertension (52.2% vs. 23.4%, *p* < 0.001), and increased overall mortality [5].

Surgical aortic valve bioprostheses were utilized in 85.4% of cases, with no significant difference observed between BEVs and SEVs. The median durations of cardiopulmonary bypass and aortic cross-clamping were similar between BEVs and SEVs. According to the EXPLANT-TAVR registry, overall intraoperative mortality was 0.8%, while in-hospital mortality was 13.0%, with no significant differences observed between BEVs and SEVs groups. Additionally, there were no statistically significant differences in the duration of mechanical ventilation, ICU stay, hospital length of stay, incidence of new pacemaker implantation, in-hospital stroke, vascular complications, and major or life-threatening bleeding events between the two cohorts [5].

At 30 days, there were no statistically significant differences observed between the BEV and SEV groups in terms of mortality (15.1% vs. 17.3%; *p* = 0.57), stroke incidence (4.4% vs. 7.1%; *p* = 0.36), or hospital readmission rates (13.9% vs. 8.9%; *p* = 0.17) [5]. Of the 247 patients who completed the one-year follow-up, the overall mortality rate was 32.8%, with comparable outcomes observed between the groups (31.8% in the BEV group versus 33.9% in the SEV group) [5].

Stroke rates were similar between the two cohorts, and no significant difference was observed in cumulative mortality over three years [5].

## 4. Technical Challenges and Expert Insights for TAVR Explantation

Explantation of transcatheter heart valves is a high-risk procedure with specific technical challenges. Patients evaluated for surgical explantation after TAVR failure are often high risk due to prior ineligibility for surgery, significant comorbidities, frailty, challenging anatomy, complex structural issues, previous operations, heavy aortic calcification, or a small aortic annulus. Emerging data—such as from Fukuhara et al.—indicate that even initially low-risk patients experience disproportionately high observed-to-expected mortality when undergoing TAVR explantation, underscoring the inadequacy of conventional risk models in this setting [11] [Figure 1].

According to the EXPLANT-TAVR registry, the majority of patients underwent concomitant procedures such as aortic root repair, ascending aortic replacement, mitral valve repair or replacement, tricuspid valve repair, and combined mitral and tricuspid surgery. These additional interventions were correlated with increased mortality rates [6]. The registry further indicated that mortality following TAV explantation was associated with longer bypass and cross-clamp times, as well as concomitant mitral or tricuspid valve interventions [5]. Previous reports have highlighted that the severe calcification and endothelialization of the transcatheter aortic valve, compounded by the calcification and degeneration of the native valve, can increase the complexity of TAV surgical explantation.

The Society of Thoracic Surgeons has recently developed updated Adult Cardiac Surgery Risk Calculators, which now incorporate advanced surgical aortic valve replacement evaluations and have been tailored to include patients with a history of prior TAVR procedures [12]. The updated STS risk score enables improved accuracy in risk prediction for this growing and high-risk patient population.

Conversely, while the EuroSCORE II risk score is widely utilized for surgical risk assessment, it does not specifically account for the unique challenges and risks associated with TAV explantation. Its predictive value for outcomes in this context is, therefore, limited and has been recognized as such.

A preoperative contrast-enhanced computed tomography (CT) scan is commonly performed to support a safe TAV explant procedure. CT scanning provides high-resolution visualization of the transcatheter heart valve, surrounding cardiac anatomy, and any anatomical variations that may influence surgical planning. Preoperative CT imaging commonly includes assessment of the aortic root and annulus dimensions, ascending aorta length, evaluation of the prosthetic valve stent frame’s relative length, and selection of optimal sites for cannulation and clamping. Additionally, this imaging modality aids in the detection of pathological conditions such as infective endocarditis involving the aortic root.

The cannulation strategy should be based on the specific TAV type and preoperative CT findings. The stent frame of the prosthetic valve is usually identified through palpation of the aorta. When palpation is not possible, epiaortic ultrasound can be used to determine the location of the frame’s borders. For supra-annular valves with frames that extend into the ascending aorta, high cannulation in the aortic arch is recommended to provide adequate space for aortic cross-clamping. The TAVR scaffold may reduce available space for placing a cannula or clamp on the ascending aorta, which can lead to the use of peripheral axillary or femoral artery cannulation as an alternative approach [3].

Standard antegrade cardioplegia is generally appropriate for intra-annular valves, where the stent frame is short. For supra-annular valves, antegrade cardioplegia cannulation should avoid the tall valve prosthesis, which can typically be palpated through the aorta. Retrograde cardioplegia is frequently utilized during TAVR-explant procedures and is typically necessary to achieve initial cardiac arrest in patients with aortic regurgitation or when access to the coronary ostia is challenging. Access to the coronary ostia prior to valve removal is often limited by the small cell openings of the stent frame [3]. Following removal of the THV, the coronary ostia are available for the administration of additional antegrade cardioplegia when required.

Left ventricular venting via the right superior pulmonary vein is typically used to enhance aortic root visualization.

For intra-annular valves, the aortotomy is performed in the standard location, using either a transverse or oblique incision. With supra-annular valves, the aortotomy may be conducted at the top of or within the frame of the valve [3,13].

Self-expanding valves are taller than balloon-expandable valves, often requiring a higher aortotomy for removal due to their larger stent frame. This can hinder visualization of the aortic annular complex and increase the risk of root damage. They also present device-specific technical challenges during explantation. Medtronic CoreValve/Evolut valves exert greater radial force and have smaller cell openings than Abbott Portico or Navitor, making access to coronary ostia more difficult, sometimes requiring cutting of the nitinol frame. Ice slushing improves nitinol frame deformability, enabling digital dissection from the aortic wall and infolding of the frame, which facilitates access to the coronary ostia and creates a plane for valve removal at the annular level. Conversely, explantation with balloon-expandable valves may be more straightforward for surgeons without TAVR experience, as their stent profile resembles surgical aortic valves. This difference may explain the higher rate of aortic root replacement during SEV explantation compared to BEV [5]. BEV explantation frequently exhibits adhesion at the aortic root, while self-expandable devices tend to adhere at the sinotubular junction; additionally, larger aortic diameters are generally associated with reduced adhesions, which facilitate dissection [14].

There is limited data comparing earlier versus later interventions. Early TAVR explantation, conducted within one month of the initial procedure, generally enables easier removal of the prosthesis because there is minimal adhesion. In contrast, delayed surgical management—especially following unsuccessful or suboptimal non-surgical approaches such as repeat TAVR—has been associated with less favorable outcomes. This may be due to factors including patient condition, increased device adherence, and the potential requirement for additional procedures [13,14].

During the TAV explant procedure, it is essential to utilize a Freer elevator to carefully access the plane between the transcatheter aortic valve frame and the aortic wall, while preserving the integrity of the aortic intima. There are established techniques designed to minimize tissue damage and reduce the risk of additional surgical interventions. The Double Kocher and Roll methods are typically employed for the explantation of balloon-expandable valves (BEVs), whereas the Tourniquet and Recapture techniques are used for the explantation of self-expanding valves (SEVs) [3,14,15].

In the Double Kocher technique, endarterectomy spatulas are used to separate the aortic wall from the distal stent frame. Once this is carried out circumferentially to halfway down the frame, the stent is secured with two long Kocher clamps. The process begins with the application of a Kocher clamp in one direction, followed by the placement of a second Kocher clamp perpendicular to the first. All sharp edges are folded inward to minimize trauma. Applying the clamps perpendicularly allows mobilization of the BEV’s sharp edge and provides a handle for valve explantation. As additional portions of the frame are separated, the clamps are repositioned further toward the base of the valve. It is essential to access the plane between the native valve leaflets and the TAVR cuff [3,14,15].

In the Roll technique, two clamps are used to grasp the valve at the commissures, positioned 180° apart, with one jaw positioned inside the transcatheter aortic valve and the other between the native valve and the transcatheter valve. Then, both clamps are rotated inward together through a complete 360° rotation [3,14,15].

In the Tourniquet technique, silk ties are passed through opposing ends of the stent frame and then looped around a section of 3/8-inch pump tubing. The ties are subsequently tightened to function as a tourniquet, facilitating the recapture of the valve [3,14,15].

While ice-cold saline irrigation may not be necessary for all nitinol frames, it can still serve as a valuable, safe adjunct in certain circumstances [12]. Detachment of the device from either native or prosthetic aortic valves is generally straightforward, as these tissues typically tolerate manipulation well [14]. However, the presence of significant calcification or abscess formation at the valve may increase the complexity of extraction procedures.

Following TAV explantation, a comprehensive assessment of the ascending aorta and aortic root is required to identify any potential injury, with aortic repair undertaken as indicated. The majority of patients undergoing TAV explantation typically require a form of aortic root reconstruction, which may include root enlargement, aortic root endarterectomy, or root replacement [16]. Root replacement is most often performed during TAVR explantation for endocarditis with periannular abscess or when a deeply endothelialized TAVR valve damages the proximal ascending aorta or sinuses during removal.

Deep implantation of valves, especially with earlier-generation transcatheter heart valves, may affect the aortomitral curtain, anterior mitral leaflet, and membranous septum. If these structures are compromised during TAVR explant, subsequent repair such as ventricular septal defect closure or concomitant mitral surgery may be necessary [5]. In cases of endocarditis, especially when accompanied by an abscess, destruction of the aortomitral curtain may require a Commando procedure to restore aortomitral continuity. Notably, patients who require concomitant surgical intervention demonstrate an elevated mortality rate of 23.8% [15].

Subsequent standard SAVR should be performed, taking into account the need for aortic annular enlargement in cases of prosthesis–patient mismatch or to facilitate future transcatheter interventions.

New generation bioprosthetic valves, such as the rapid deployment Intuity Elite and sutureless aortic bioprosthetic valves like Perceval S, may significantly facilitate the TAV explant procedure. These valves provide the benefits of reduced aortic cross-clamp time and shorter extracorporeal circulation duration and are likely associated with fewer postoperative complications compared to traditional bioprosthetic valves [16,17]. On the other hand, they have higher pacemaker implantation and equivalent or higher perivalvular leak rates than standard surgical valves [16]. These surgical valves are recommended for elderly and frail patients who have comorbidities and a higher likelihood of complications related to surgery. Sutureless aortic bioprosthetic valves provide the benefit of a larger effective orifice area, reducing the incidence of patient–prosthesis mismatch and allowing surgeons to avoid complex aortic root procedures [17].

## 5. The SURPLUS Hybrid Procedure

Surgical Resection of Prosthetic Valve Leaflets Under Direct Vision (SURPLUS) is a hybrid, less invasive approach in which the transcatheter aortic valve leaflets are surgically excised while the frame of the valve remains intact, followed by direct implantation of a balloon-expandable valve under both direct visualization and fluoroscopic guidance. The procedure is typically conducted through an upper mini sternotomy with peripheral cannulation or, alternatively, via a standard median sternotomy with central cannulation and cardioplegic arrest.

This approach poses fewer technical challenges than conventional surgical transcatheter valve explantation. In particular, it reduces excessive aortic wall manipulation and decreases the risk of concomitant cardiac procedures, which may be associated with reduced operative mortality. It also reduces bypass, cross-clamp, and procedure times, which may contribute to improved postoperative outcomes. Additionally, it enables proper alignment of the commissures of the two transcatheter heart valves, which allows future coronary access [18].

SURPLUS is generally indicated for elderly or high-risk patients with degenerated self-expandable transcatheter heart valves who are eligible for open-heart surgery. It is not indicated when explantation is required due to infective endocarditis, as complete removal of the infected transcatheter heart valve (including its frame) is necessary. Similarly, it may be unsuitable in cases of severe paravalvular leak or prosthesis–patient mismatch [3,18].

The SURPLUS technique offers increased efficiency and simplicity, with encouraging preliminary outcomes; however, additional data collection and extended validation are required to substantiate these findings.

Collaboration between surgical and interventional teams is essential to ensuring optimal procedural outcomes, as the complexity of individual cases often requires a dynamic, multidisciplinary approach. This highlights the ongoing need for continual advancement of surgical techniques and patient management strategies.

## 6. Discussion

Surgical explantation of transcatheter aortic valves, even in low-risk patients, can result in significant morbidity and mortality. Although it occurs infrequently, this procedure has reported 30-day mortality rates between 15% and 20%, with higher rates observed when concomitant procedures or endocarditis are involved.

On the other hand, the TAVR technique provides a less invasive option for treating patients with failed transcatheter aortic bioprostheses, although it is associated with higher rates of valve malposition, coronary obstruction, and patient–prosthesis mismatch. It is considered a therapeutic alternative for selected patients, particularly those with structural valve degeneration, low risk of coronary obstruction, and the potential for favorable hemodynamic outcomes. However, this approach may not be appropriate for all TAVR recipients due to anatomical factors or the presence of endocarditis. The TAVR procedure is linked to a higher rate of reintervention; however, it is associated with reduced technical complexity and lower 30-day mortality compared to surgical reoperation [19].

Recent Medicare data show an association between surgical explant and improved long-term survival when compared to repeated transcatheter procedures. At the NY Valves 2025 symposium, Fukuhara presented an analysis of 4443 Medicare beneficiaries who underwent TAVR and later received either redo TAVR (*n* = 2553) or TAVR explantation (*n* = 1890) [20]. The 30-day mortality rate was higher for the TAVR-explant group compared to the redo TAVR group (15.3% vs. 4.9%). In a propensity score-matched analysis including 1584 patients in each group, survival rates were higher in the TAVR-explant arm than in redo TAVR, with survival curves intersecting at 1.8 years (*p* < 0.001). The risk associated with TAVR explantation dropped below that of redo TAVR in 7 months (HR 0.86; 95% CI 0.77–0.97). Additionally, fewer patients who underwent TAVR explantation required a second reintervention compared to those who had redo TAVR (6.2% vs. 31.2%; *p* = 0.01). Stratification by age showed that explantation conferred a significant survival advantage over redo TAVR in individuals aged 65–70 years (beginning at 1.3 years; *p* = 0.011), while no survival benefit was observed for those aged over 70 years [20]. The study has some limitations, and a well-designed RCT in this area would be challenging. Ongoing data collection and collaborative research efforts will be crucial in addressing current knowledge gaps.

The lack of standardized protocols and established guidelines for managing TAVR explantation presents a significant clinical challenge. Currently, there is limited published data regarding surgical explantation of transcatheter aortic valves. Further studies may help refine surgical strategies and develop evidence-based protocols for this clinical scenario. Prospective multicenter registries could provide additional data on long-term outcomes after TAVR explantation and contribute to the development of standardized surgical approaches [Figure 1].

**Figure 2:**
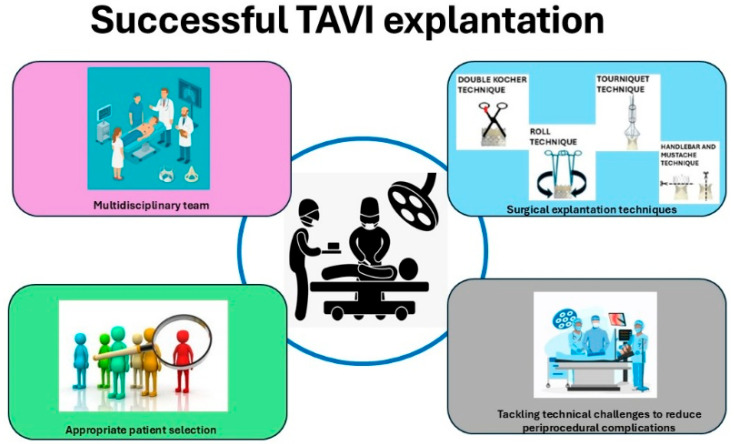
Successful surgical transcatheter aortic valve explantation.

A multidisciplinary strategy that incorporates the expertise of cardiac surgeons, interventional cardiologists, infectious disease specialists, and radiologists is critical to enhancing diagnosis, management, and postoperative outcomes for this high-risk patient group. Through the integration of multiple clinical viewpoints, the healthcare team is well positioned to address the distinct complexities associated with each case, such as anatomical variances, device-related considerations, and comorbidities [Figure 2].

Careful patient selection and prompt intervention remain essential to improve outcomes in this challenging patient population. Comprehensive preoperative planning utilizing CT can minimize the likelihood of iatrogenic injury and streamline procedural execution. In addition, regular post-TAVR monitoring and timely referral to surgical teams are advised to avoid interventions in patients presenting with advanced pathology [3] [Figure 1 and Figure 2].

When TAVR explantation is indicated, it is essential for surgeons to be aware of the complex challenges associated with different TAVI device designs, sizes, and the specific anatomical considerations of each patient. A patient-tailored approach is associated with improved surgical outcomes. A thorough understanding of TAVR system characteristics and their interactions with native anatomical structures is required.

Standardizing surgical THV explantation techniques may reduce complications while improving both safety and reproducibility. Furthermore, such standardization facilitates a more efficient learning process. Collaborative networks between surgical centers and referring institutions can support the exchange of experience and may assist in refining best practices. Restricting TAVR-explant procedures to high-volume institutions and centers with established expertise may be associated with lower morbidity and mortality rates.

Therefore, when considering transcatheter aortic valve implantation for younger patients with aortic stenosis, clinicians should recognize that subsequent surgical removal and replacement of an infected or degenerative transcatheter prosthesis may pose significant technical difficulties and risks. The explantation of TAV devices is associated with notable surgical hazards that must be carefully weighed in lifetime management of aortic stenosis. Especially in younger and lower-risk patients with aortic stenoses, it is important to individualize the risk-to-benefit ratio of TAVR, particularly when minimally invasive aortic valve surgery presents a safe alternative to conventional aortic valve replacement. It is essential that patients being evaluated for transcatheter aortic valve implantation are comprehensively informed regarding potential future reinterventions, including the possibility of requiring a TAV explant procedure.

## 7. Conclusions

Over the next decade, a rise in complex aortic valve reoperations following TAVR procedures is expected. Clinical assessment and multidisciplinary collaboration are key for selecting appropriate TAVR candidates, with individualized risk–benefit evaluation. Heart teams are advised to clearly communicate potential risks of reoperation during shared decision making with patients. Outcomes following TAVR explants could improve among lower-risk patients with careful monitoring, timely referrals, preoperative planning, patient-tailored approaches, and standardized TAVR-explant techniques. Further large-scale studies are required to optimize surgical approaches and develop robust, evidence-based protocols for managing this complex clinical situation.

## Figures and Tables

**Table 1:** The EXPLANT-TAVR registry distinguishes between balloon-expandable and self-expanding valves in terms of explantation reasons.

Indication	SEV (%)	BEV (%)	*p*-Value
Endocarditis	36.0	55.4	<0.001
Paravalvular leak	21.2	11.9	0.014
Structural valve deterioration	30.2	21.8	0.065
Prosthesis–patient mismatch	8.5	10.4	0.61

**Table 2:** The EXPLANT-TAVR registry showed that the SEVs had fewer urgent or emergency surgeries and more root replacements.

Group	Urgent/Emergency Surgeries (%)	*p*-Value (Urgent/Emergency)	Root Replacement (%)	*p*-Value (Root Replacement)
SEV	52.0	0.057	15.3	0.016
BEV	62.3	0.057	7.4	0.016

## Data Availability

Data sharing is not applicable.

## Abbreviations

The following abbreviations are used in this manuscript:

TAV	transcatheter aortic valve
TAVR	transcatheter aortic valve replacement
BEV	balloon-expandable valve
SEV	self-expanding valve
STS	Society of Thoracic Surgeons
ACC	American College of Cardiology
CT	Computed Tomography

## References

[1] Thourani V.H., Leon M.B., Makkar R., Ascione G., Szeto W.Y., Madhavan M.V., Kodali S.K., Hahn R.T., Pibarot P., Malaisrie S.C. (2025). Five-Year Outcomes in Low-Risk Patients Undergoing Surgery in the PARTNER 3 Trial. Ann. Thorac. Surg..

[2] Forrest J.K., Yakubov S.J., Deeb G.M., Gada H., Mumtaz M.A., Ramlawi B., Bajwa T., Crouch J., Merhi W., Wai Sang S.L. (2025). 5-Year Outcomes After Transcatheter or Surgical Aortic Valve Replacement in Low-Risk Patients with Aortic Stenosis. J. Am. Coll. Cardiol..

[3] Kaneko T., Bapat V.N., Alakhtar A.M., Zaid S., George I., Grubb K.J., Harrington K., Pirelli L., Atkins M., Desai N.D. (2025). Transcatheter heart valve explantation for transcatheter aortic valve replacement failure: A Heart Valve Collaboratory expert consensus document on operative techniques. J. Thorac. Cardiovasc. Surg..

[4] Durand E., Eltchaninoff H., Tchetche D., Levesque T., Garmendia C., Iung B., Benamer H., Cayla G., Van Belle E., Commeau P. (2025). Predictors of Outcomes of Reintervention After Transcatheter Aortic Valve Replacement: FRANCE 2 and FRANCE TAVI Registries. J. Am. Coll. Cardiol..

[5] Zaid S., Kleiman N.S., Goel S.S., Szerlip M.I., Mack M.J., Marin-Cuartas M., Mohammadi S., Nazif T.M., Unbehaun A., Andreas M. (2024). Impact of transcatheter valve type on out-comes of surgical explantation after failed transcatheter aortic valve replacement: The EXPLANT-TAVR international registry. EuroIntervention.

[6] Bapat V.N., Zaid S., Fukuhara S., Saha S., Vitanova K., Kiefer P., Squiers J.J., Voisine P., Pi-relli L., von Ballmoos M.W. (2021). Surgical Explantation After TAVR Failure: Mid-Term Outcomes From the EXPLANT-TAVR International Registry. JACC Cardiovasc. Interv..

[7] Pineda A.M., Harrison J.K., Kleiman N.S., Rihal C.S., Kodali S.K., Kirtane A.J., Leon M.B., Sherwood M.W., Manandhar P., Vemulapalli S. (2019). Incidence and Outcomes of Surgical Bailout During TAVR: Insights From the STS/ACC TVT Registry. JACC Cardiovasc. Interv..

[8] Malvindi P.G., Luthra S., Sarvananthan S., Zingale A., Olevano C., Ohri S. (2021). Surgical treatment of transcatheter aortic valve infective endocarditis. Neth. Heart J..

[9] Calcaterra D., Harris K., Goessl M., Dasari G., Kaur N., Chavez I. (2021). Findings of prosthetic valve endocarditis in the balloon-expandable trans-catheter aortic valve: Review of the literature and tips of management. J. Cardiothorac. Surg..

[10] Pisani A., Hounat F., Brega C., Borghese O., Braham W., Alkhoder S. (2020). Infective endocarditis following transcatheter aortic valve implantation. Ann. Cardiol. Angeiol..

[11] Fukuhara S., Suzuki T., Deeb G.M., Ailawadi G., Patel H.J., Yang B. (2025). Clinical outcomes of TAVR explant stratified by original risk profile: Insights from 110 TAVR explants. Ann. Cardiothorac. Surg..

[12] Τhe Society of Thoracic Surgeons SAVR After TAVR Risk Calculator. https://www.sts.org/resources/savr-after-tavr-risk-calculator.

[13] Sakakura R., Fukuzawa M., Sugiyama H., Tani K., Yoshida T., Murakami A., Terai H., Ueyama K. (2025). Early surgical explantation of a TAVI valve for severe hemolytic outcomes caused by mild paravalvular leak. Gen. Thorac. Cardiovasc. Surg. Cases.

[14] Fukuhara S. (2020). Safe late explantation of transcatheter aortic bioprosthesis. Ann. Thorac. Surg..

[15] Kurosaka K., Iino K., Yamamoto Y., Takemura H. (2025). Recapture technique for the surgical explantation of an infected self-expanding prosthesis after transcatheter aortic valve re-placement prior to surgical aortic valve replacement. Interdiscip. Cardiovasc. Thorac. Surg..

[16] Di Eusanio M., Berretta P. (2020). The sutureless and rapid-deployment aortic valve replacement international registry: Lessons learned from more than 4,500 patients. Ann. Cardiothorac. Surg..

[17] Androutsopoulou V., Zotos P.A., Xanthopoulos A., Boultadakis E., Magouliotis D., Schizas N., Iliopoulos D.C., Skoularigis J., Athanasiou T. (2025). Individualized Selection of Valve Intervention Strategies in Aortic Disease Is Key for Better Outcomes. J. Pers. Med..

[18] Bcharah G., Zhuang J., Farina J.M., Jenkins J.A., Sell-Dottin K.A. (2025). TAV-in-TAV Explant Through Surgical Resection of Prosthetic Valve Leaflets Under Direct Vision: SURPLUS. Methodist Debakey Cardiovasc. J..

[19] Fukuhara S., Kim K.M., Yang B., Romano M., Ailawadi G., Patel H.J., Deeb G.M. (2024). Reoperation following transcatheter aortic valve replacement: Insights from 10 years’ experience. J. Thorac. Cardiovasc. Surg..

[20] Fukuhara S. TAVR reintervention strategies: Unveiling trends and outcomes of redo TAVR and TAVR explant. Proceedings of the New York Valves 2025.

